# Predictive Ability of Combined Factor Scores for Chromosomal Abnormalities in Pregnant Women With Polyhydramnios

**DOI:** 10.7759/cureus.33106

**Published:** 2022-12-29

**Authors:** Genichiro Sotodate, Manami Akasaka, Atsushi Matsumoto, Yukiko Toya, Nao Takashimizu, Shigekuni Tsuchiya

**Affiliations:** 1 Department of Pediatrics, Iwate Medical University, Yahaba, JPN

**Keywords:** prenatal factor, pregnancy, predictive ability, polyhydramnios, chromosomal abnormalities

## Abstract

Aim: This study investigated factors that can predict chromosomal abnormalities in pregnant women with polyhydramnios. The ability of prenatal factors to predict chromosomal abnormalities was evaluated using receiver operator characteristic curves.

Methods: Of 76 eligible pregnant women, major anomalies were detected in 41 (54%) and chromosomal abnormalities in 19 (25%): trisomy 13 in one, trisomy 18 in 10, trisomy 21 in seven, and 22q11.2 deletion syndrome in one. Combined factor scores, including maternal age, major anomaly, abdominal circumference percentile, femur length percentile, and estimated fetal weight percentile, proved to be good predictors (area under the curve, 0.81-0.87) of chromosomal abnormalities and showed a sensitivity of 79% and specificity of 75%.

Conclusion: Combined scores demonstrated more accuracy than individual factors for predicting chromosomal abnormalities. Even if an anomaly is not detected on fetal ultrasonography, in cases with higher scores, chromosomal abnormalities should be suspected, and delivery at a level III facility may be recommended.

## Introduction

Polyhydramnios is an excessive amount of amniotic fluid in pregnancy. Polyhydramnios occurs in 0.5%-2% of all pregnancies [1-5]. In pregnancies diagnosed with polyhydramnios, 60%-70% of cases are idiopathic with no identified underlying cause, while 30%-40% are due to a congenital anomaly or maternal disease [6,7]. Previous studies on polyhydramnios in pregnancy have reported that the fetal anomaly or chromosomal abnormality prevalence rates range from 4.2% to 38% [1-6,8,9].

Women with polyhydramnios during pregnancy should undergo a targeted ultrasonographic examination to evaluate for the presence of fetal anomalies. Fetal growth restriction (FGR) is associated with a high risk of underlying fetal abnormality, including trisomy 13 or 18 [2,9-11]. Pregnant women with severe polyhydramnios are recommended to deliver at tertiary centers where obstetric and pediatric support is available at delivery, given the significant possibility that fetal anomalies may be present [10]. Therefore, this study investigated factors that can predict chromosomal abnormalities in pregnant women with polyhydramnios.

## Materials and methods

This retrospective cohort study was conducted at the Iwate Medical University in Japan. Pregnant women with polyhydramnios admitted to our center for treatment between April 2012 and March 2021 were included. Pregnant women with diabetes or multiple pregnancies were excluded because of their disproportionate risk of polyhydramnios caused by factors other than a fetal anomaly. We collected all data from medical records and ultrasonography reports of the patients. The following maternal data were collected from the medical records: maternal age, amniocentesis, and fetal death. The following ultrasound data were collected: degree of polyhydramnios, percentile of estimated fetal weight (EFW), biparietal diameter (BPD), abdominal circumference (AC), and femur length (FL). The following neonatal data were collected: gestational age at birth, birth weight, small-for-gestational-age (SGA) (<10th percentile: <10%ile), anomaly type (major or minor), chromosomal abnormalities, and postnatal death.

The obstetricians perform ultrasonography themselves or review ultrasonography performed by a sonographer. Polyhydramnios was defined as an amniotic fluid index (AFI) ≥24 cm or single deepest vertical pocket (SDP) ≥8 cm [5,10]. The degrees of polyhydramnios were divided into the following: mild, defined as an AFI of 24-29 cm or an SDP of 8-11 cm; moderate, defined as an AFI of 30-34 cm or an SDP of 12-15 cm; and severe, defined as AFI ≥35 cm or an SDP >16 cm [5]. Fetal ultrasonographies were performed every two to four weeks. Periods of diagnosis were subdivided into the following: early, defined as 20-29 gestational weeks; medium, defined as 30-34 gestational weeks; and late, defined as ≥35 gestational weeks. If multiple fetal ultrasonographies were performed in the same period, a percentile value was taken off the median of the measurement values obtained in that period. We measured EFW, BPD, AC, and FL of the 10th percentile or less of the mean value for gestational age according to the Japanese standard curve [12]. A fetus in <10th percentile (<10%ile) at least once in a given period was categorized as being in this percentile. An anomaly was considered major if it was potentially life-threatening, required medical or surgical treatment in the immediate neonatal period, or involved a congenital heart disease [5]. Investigations for major anomalies included medical examination, radiography, and ultrasonography, routinely performed in the immediate neonatal period by attending faculty pediatricians; karyotype analysis was performed at the neonatologist’s discretion. In cases of fetal death, a major anomaly was considered present if detected during the antepartum period, at delivery, or upon necropsy. Neonatal death was defined as death before discharge from the hospital.

Continuous variables are expressed as mean ± standard deviation. Categorical variables are expressed as the number and percentage of the total. Statistical analyses for baseline group comparisons were performed with the chi-squared or Fisher’s exact test for categorical variables and the Mann-Whitney U test for continuous variables. The area under the receiver operating characteristic curve (AUC) was calculated to assess the predictive ability of chromosomal abnormalities. The AUC results were considered excellent, good, fair, poor, or failed for AUC values of 0.9-1, 0.8-0.9, 0.7-0.8, 0.6-0.7, and 0.5-0.6, respectively. Data were analyzed using SPSS version 23 (IBM Corp., Armonk, NY, USA). Statistical significance was set at p <0.05.

Our study adhered to ethical guidelines and was approved by our institutional ethics committee (approval number, MH2022-013). Informed consent was obtained from the parents of infants/children using an opt-out approach.

## Results

Overall, 76 pregnant women with polyhydramnios met our inclusion criteria. Of these, three fetuses died in utero. A major anomaly was detected in 41 of 76 infants (54%), and 13 of these infants had multiple major anomalies. Among the 41 infants with anomalies, 54 major anomalies were detected (Table 1).

**Table 1:** List of major anomalies (n=54) present in the 41 included infants

Central nervous system		Skeletal	
Cerebellar hypoplasia	3	Asphyxiating thoracic dysplasia	1
Agenesis of corpus callosum	1	Thanatophoric dysplasia	1
Spina bifida	1	Chondrodysplasia punctata	1
Thorax		Gastrointestinal	
Diaphragmatic hernia	3	Duodenal atresia	9
Tracheal atresia	3	Esophageal atresia	6
Hypoplastic lung	2	Midgut volvulus	2
Pulmonary airway malformation	1	Intestinal atresia	1
Cardiac		Imperforate anus	1
Ventricular septal defect	9	Dysplasia of the oral cavity	1
Double outlet right ventricle	5		
Tetralogy of Fallot	2		
Atrioventricular septal defect	1		

The prevalence rate of a major anomaly increased with increasing severity of polyhydramnios; however, there was no significant difference (p=0.09). Nineteen of the 76 infants had chromosomal abnormalities (trisomy 13 in one, trisomy 18 in 10, trisomy 21 in seven, and 22q11.2 deletion syndrome in one). Polyhydramnios with chromosomal abnormalities was significantly associated with older maternal age, lower birth weight, higher incidence of intrauterine fetal death, EFW <10th percentile, AC <10th percentile, AC <3rd percentile, FL <10th percentile, major anomaly, SGA, and neonatal death (Table 2).

**Table 2:** Patient characteristics EFW: estimated fetal weight; BPD: biparietal diameter; AC: abdominal circumference; FL: femur length; SGA: small for gestational age; %ile: percentile.

Characteristic		Total (N=76)	Chromosomal abnormalities		p-Value
			Yes (N=19)	No (N=57)	
Maternal					
Maternal age	Years	32.8±5.4	35.8±5.2	31.8±5.5	<0.05
	≤29	21	1	20	<0.05
	30–39	43	11	32	
	≥40	12	7	5	
Degree of polyhydramnios	Mild	30	6	24	0.708
	Moderate	15	4	11	
	Severe	31	9	22	
Amniocentesis		25	9	16	0.121
Fetal death		3	3	0	<0.05
Fetal ultrasonography					
EFW	<10%ile	18	10	8	<0.05
	<3%ile	7	4	3	0.061
BPD	<10%ile	3	2	1	0.152
	<3%ile	2	2	0	0.06
AC	<10%ile	14	8	6	<0.05
	<3%ile	8	6	2	<0.05
FL	<10%ile	23	11	12	<0.05
	<3%ile	13	6	7	0.077
Neonatal					
Gestational age		37.2±2.1	36.7±1.7	37.3±2.2	0.472
Birth weight		2750.1±688.7	2302.2±609.3	2886.0±657.4	<0.05
SGA		15	8	7	<0.05
Major anomaly		41	18	23	<0.05
Postnatal death		19	11	8	<0.05

We produced a combined factor score applicable to pregnant women with polyhydramnios using a calculation involving five variables (maternal age, major anomaly, EFW percentile, AC percentile, and FL percentile) for the prediction of chromosomal abnormalities (Table 3).

**Table 3:** Combined factor score for the prediction of chromosomal abnormalities using a calculation involving five variables EFW: estimated fetal weight; AC: abdominal circumference; FL: femur length; %ile: percentile.

Factor		Score		
		0	1	2
Maternal age	Years	≤29	30-39	≥40
Major anomaly		0	1	≥2
EFW	%ile	>10	3-10	<3
AC	%ile	>10	3-10	<3
FL	%ile	>10	3-10	<3

Each variable was attributed 0-2 points. For example, a case with a maternal age of 35 years, AC in the 5th percentile, and one major anomaly detected would be attributed a factor score of 3 points. Table 4 shows the AUC for the predictive ability of chromosomal abnormalities.

**Table 4:** Area under the receiver operating characteristic curve for the predictive ability of the variables for chromosomal abnormalities AUC: area under the receiver operating characteristic curve; CI: confidence interval; MA: maternal age; EFW: estimated fetal weight; AC: abdominal circumference; FL: femur length.

Factor	AUC	95% CI
MA	0.73	(0.60-0.85)
EFW	0.69	(0.55-0.84)
FL	0.69	(0.39-0.70)
AC	0.67	(0.51-0.82)
Anomaly	0.80	(0.69-0.90)
MA+anomaly	0.86	(0.78-0.94)
MA+EFW	0.81	(0.70-0.93)
MA+AC+FL	0.81	(0.69-0.93)
EFW+anomaly	0.81	(0.71-0.92)
MA+AC+FL+anomaly	0.87	(0.79-0.95)
MA+EFW+anomaly	0.86	(0.78-0.94)

Maternal age, major anomaly, EFW percentile, AC percentile, and FL percentile alone appeared to be poor or fair predictors (AUC 0.67-0.80) of chromosomal abnormalities. Combined factor scores appeared to be good predictors (AUC 0.81-0.87) of chromosomal abnormalities, and their predictive abilities were superior to those of individual factors. Using a combined factor score that included maternal age, major anomaly, AC percentile, and FL percentile, a cutoff score of 3 points was associated with a sensitivity of 79% and a specificity of 75% for the prediction of chromosomal abnormalities (AUC, 0.87; 95% confidence interval [CI], 0.79-0.95). Using a combined factor score including maternal age, major anomaly, and EFW percentile, a cutoff score of 3 points was associated with a sensitivity of 79% and a specificity of 75% for the prediction of chromosomal abnormalities (AUC, 0.86; 95% CI, 0.78-0.94).

## Discussion

This study showed that combined factor scores including maternal age, major anomaly, EFW percentile, AC percentile, and FL percentile demonstrated good predicting ability for chromosomal abnormalities.

FGR is observed in approximately 3%-6% of polyhydramnios cases, and the finding of polyhydramnios with FGR is of great concern in terms of the risk of underlying major fetal anomalies, chromosomal abnormalities, or both [2,7,9,11]. A multivariable logistic regression analysis of 208 pregnant women with polyhydramnios found that the severity of polyhydramnios and reduction of fetal movement are independently associated with chromosomal or genetic disorders. However, the presence of a major structural defect was not associated with chromosomal or genetic disorders [4]. A multivariate analysis of 5,043 pregnant women who underwent amniocentesis revealed that cystic hygroma, congenital heart disease, and abnormal extremities were associated with fetal chromosomal abnormalities, but FGR was not associated with fetal chromosomal abnormalities [13]. Hara et al. [11] found that a significant risk of fetal anomaly should be considered in pregnant women with severe polyhydramnios and FGR or those requiring prenatal treatment.

The Society for Maternal-Fetal Medicine recommends women be offered fetal diagnostic testing, including chromosomal microarray analysis, when FGR is detected and fetal malformation, polyhydramnios, or both are also present [14]. The combination score had a higher AUC and was more effective than individual factors for predicting chromosomal abnormalities, indicating that the combined score is useful for predicting chromosomal abnormalities.

In pregnant women with polyhydramnios, the incidence rate of a major anomaly detected with prenatal ultrasonography ranges from 80% to 90% [2,6,9]. If no anomaly is identified prenatally with fetal ultrasonography in pregnant women with severe polyhydramnios, then anomalies and chromosomal abnormalities, which are difficult to detect with ultrasonography and screening examinations, cannot be ruled out [2,10]. We reported that infants with congenital heart disease born in level I (practicing obstetrics and gynecology clinics) and level II facilities (regional general hospitals) were more likely to be diagnosed late, owing to the inexperience of the clinic/hospital personnel with congenital heart disease [15]. In level I and II facilities, referral to a higher-level facility might be delayed for pregnant women with polyhydramnios and no fetal anomaly. Using a combined score including maternal age and EFW percentile, a cutoff of 2 points showed a sensitivity of 79% and a specificity of 79% for the prediction of chromosomal abnormalities (AUC, 0.81; 95% CI, 0.70-0.93). Even if an anomaly is not detected on fetal ultrasonography, in cases with higher scores, chromosomal abnormalities should be suspected, and delivery at a tertiary facility may be recommended owing to the risk of maternal and neonatal complications. For obstetricians and pediatricians, future efforts need to join forces to incorporate combined scores into an algorithm for chromosomal abnormalities detection in relatively underserved regions.

Chromosomal abnormalities increase with increasing maternal age. The frequency of chromosomal abnormalities has been reported at 0.19%-0.24% in pregnant women aged 20-29 years, 0.26%-1.24% in women aged 30-39 years, and 1.58%-14.9% in women aged 40-49 years [16]. In the current study, these frequencies among pregnant women with polyhydramnios were 4.7% in women aged 20-29 years, 25.6% in women aged 30-39 years, and 58.3% in women aged 40-49 years. This suggests that women with polyhydramnios are 10 times more likely to have chromosomal abnormalities than women without polyhydramnios in the same age group.

This study had some limitations. A relatively small number of infants were included, and the study was conducted at a single institution. There were more cases of severe polyhydramnios than of moderate polyhydramnios. Two hospitals perform surgery on neonates in our region, and our hospital is the only facility with a neonatal intensive care unit; hence, more severe cases may have been referred to our hospital. Our study population might not reflect the characteristics of pregnant women with polyhydramnios observed elsewhere. Given the small number of chromosomal abnormalities, the validity of the combined factor score could not be assessed according to each chromosomal abnormality.

## Conclusions

Combined scores, including maternal age, major anomaly, AC percentile, and FL percentile, showed higher AUCs and were more accurate than individual factors (0.81-0.87 vs. 0.67-0.80) for predicting chromosomal abnormalities. Infants with chromosomal abnormalities born in level I and level II facilities might be more likely to be diagnosed late, owing to the inexperience of the clinic/hospital personnel with chromosomal abnormalities. The combined factor scores are useful especially for level I and II facilities.

In cases with higher scores, chromosomal abnormalities should be suspected, even if an anomaly is not detected on fetal ultrasonography, and delivery at a level III facility may be recommended owing to the risk of maternal and neonatal complications.
